# The “Direct Structural Disruption” Hypothesis: *Bacteroides fragilis* Toxin as a Potentiating Cofactor in MASH Pathogenesis

**DOI:** 10.3390/toxins18050200

**Published:** 2026-04-24

**Authors:** Ju-Eun Hong, Soonjae Hwang

**Affiliations:** 1 Department of Biomedical Laboratory Science, College of Software Digital Healthcare Convergence, Yonsei University MIRAE Campus, Wonju 26493, Republic of Korea; jehong@yonsei.ac.kr; 2 Yonsei Institute of Pharmaceutical Sciences, College of Pharmacy, Yonsei University, Incheon 21983, Republic of Korea; 3 G-FRIS (Green-Futures Research Institute of Science), G-LAMP, Yonsei University, Seoul 03722, Republic of Korea

**Keywords:** metabolic dysfunction-associated steatohepatitis (MASH), enterotoxigenic *Bacteroides fragilis* (ETBF), *Bacteroides fragilis* toxin (BFT), structural disruption, gut–liver axis, hepatic stellate cells (HSCs)

## Abstract

Metabolic dysfunction-associated steatohepatitis (MASH) is a complex, multifactorial disease heavily influenced by the gut–liver axis. While enterotoxigenic *Bacteroides fragilis* (ETBF) and its principal virulence factor, *B. fragilis* toxin (BFT)—a zinc-dependent metalloprotease—are well-known for disrupting intestinal barriers, their potential systemic impact on distant organs remains an emerging area of interest. Although various gut-derived factors contribute to hepatic inflammation, the precise molecular triggers that exacerbate the transition from simple steatosis to progressive fibrosis remain incompletely understood. This review proposes the “Direct Structural Disruption” hypothesis, examining the biological activity of BFT and its proposed role in MASH pathogenesis. We postulate that under permissive conditions, systemic BFT may target hepatic structural proteins (e.g., cadherins). This hypothesized architectural impairment amplifies canonical fibrogenic signaling and hepatic stellate cell (HSC) activation. In addition, we discuss current challenges in the detection and characterization of systemic BFT, particularly the technical limitations in clinical diagnostics stemming from its profound structural homology with host metalloproteinases. Future research integrating advanced diagnostic methodologies and liver-specific in vivo models is essential to elucidate these pathophysiological mechanisms and evaluate the ETBF-BFT axis as a complementary target in progressive MASH.

## 1. Introduction

The global prevalence of Metabolic Dysfunction-Associated Steatohepatitis (MASH) has surged in tandem with the obesity epidemic, positioning it as a leading cause of end-stage liver disease and hepatocellular carcinoma (HCC) [1,2]. Despite its clinical significance, MASH remains a pathologically heterogeneous condition. The transition from simple steatosis to progressive fibrosis—often termed the “point of architectural non-return”—varies significantly among individuals. While many patients maintain a stable clinical profile for decades, a subset experiences accelerated fibrotic remodeling [3]. This clinical divergence suggests that metabolic drivers alone tell only half the story; there must be a potent exogenous accelerator capable of exacerbating the hepatic microenvironment.

For years, the “multiple-hit” hypothesis has provided the primary framework for understanding MASH, emphasizing the interplay between lipotoxicity, oxidative stress, and gut-derived endotoxemia [4]. Central to this paradigm is the translocation of Lipopolysaccharide (LPS) via the gut–liver axis, triggering inflammation through Toll-like receptor 4 (TLR4) [5]. However, while the LPS-TLR4 axis explains chronic low-grade inflammation, we propose that it could be complemented by a “structural hit”—an enzymatic trigger capable of destabilizing the junctional complexes that preserve liver architecture.

We propose that enterotoxigenic *Bacteroides fragilis* (ETBF) and its primary virulence factor, the *B. fragilis* toxin (BFT), represent this missing link. BFT, also known as fragilysin, is a 20 kDa zinc-dependent metalloprotease with a unique biochemical signature: the highly specific proteolytic cleavage of the extracellular domain of E-cadherin [6]. While ETBF is a well-documented driver of colorectal cancer [7], emerging evidence suggests its pathological influence is not confined to the gut. Notably, recent studies in murine models have demonstrated that BFT can translocate systemically and remain detectable in the circulation for weeks, acting as a persistent systemic effector [8]. However, direct evidence of circulating BFT in humans remains limited.

In the context of MASH, we postulate that BFT breaches the intestinal barrier and rides the portal circulation to the liver. Upon entry into the hepatic microenvironment, BFT is hypothesized to initiate a compounding cascade with metabolic toxins. First, it could target endothelial and hepatocyte adherens junctions, potentially triggering cell death and polarity loss. Second, and more pivotally, BFT’s compact molecular weight allows it to penetrate hepatic sinusoidal fenestrae—structures functionally compromised by MASH-driven hemodynamic stress [9]—to reach quiescent hepatic stellate cells (HSCs). By the proposed cleavage of junctional proteins on the HSC surface, BFT may effectively “unlock” membrane-bound β-catenin, activating mechanotransduction pathways (e.g., Hippo-YAP) and driving a fibrogenic program independent of classical cytokine signaling [10,11].

This review introduces the “Direct Structural Disruption Hypothesis.” By reframing MASH progression as a consequence of toxin-mediated proteolytic failure rather than just secondary inflammation, we aim to provide a new mechanistic basis for rapid fibrosis and identify the ETBF-BFT axis as a precise therapeutic target.

## 2. ETBF as a Commensal Pathobiont: Context-Dependent Pathogenesis in MASH

*Bacteroides fragilis* is a predominant obligate anaerobe within the human colonic microbiota [12]. However, the enterotoxigenic strain (ETBF), harboring the *bft* gene, functions as a pathobiont—a resident that becomes pathogenic under specific host triggers. While asymptomatic carriage is common (10–35%) [13], the progression to systemic sequelae like liver injury appears highly selective. We hypothesize that the transition of BFT from a localized secretome to a systemic driver of MASH requires specific restrictive conditions.

First, the physical translocation of BFT into the portal circulation is a prerequisite for direct hepatic injury. While BFT inherently possesses the proteolytic capacity to cleave intestinal E-cadherin and compromise the mucosal barrier [14], we hypothesize that this process is powerfully amplified in the MASH microenvironment. The underlying metabolic stress (e.g., lipotoxicity and altered bile acids) inherently weakens junctional integrity [15], acting in tandem with BFT’s enzymatic activity. This compounded barrier disruption facilitates the massive escape of BFT from the intestinal lumen into the systemic circulation.

Second, while highly speculative, we further hypothesize that host immune tolerance may play a critical role in facilitating systemic pathogenesis. BFT belongs to the metzincin superfamily and shares remarkably high structural homology with mammalian ADAM and MMP families [16]. Because of this profound molecular mimicry, the host immune system could theoretically exhibit tolerance, resulting in impaired neutralizing antibody responses. Similar immune evasion strategies via molecular mimicry have been documented in other bacterial and viral pathogens [17,18,19]. Patients presenting with both a severely compromised gut barrier and a blunted humoral immune response against BFT would be uniquely vulnerable to its systemic effects.

### 2.1. Mechanisms of Pathological Translocation to the Hepatic Parenchyma

The flow of gut-derived microbial products to the liver is a well-established driver of MASH progression. Historically, this gut–liver axis has been predominantly characterized by the “Indirect Inflammatory Hit” model, centered on endotoxins such as LPS [5]. In this classical paradigm, translocated LPS triggers chronic inflammation primarily via TLR4 activation on Kupffer cells [4].

While this immune-mediated pathway is crucial for establishing chronic low-grade inflammation, we hypothesize that it functions in tandem with a “Direct Structural Hit” in more progressive phenotypes. Unlike LPS, which primarily acts as an indirect inflammatory signaling molecule, BFT functions as a distinct catalytic effector. Upon reaching the portal circulation, BFT utilizes its metalloprotease activity to directly target and induce the proteolytic disassembly of junctional complexes within the liver’s architecture [20].

Rather than bypassing immune mediation, we propose that this enzymatic disruption acts in tandem with existing inflammatory pathways. Through the enzymatic cleavage of cadherin-based adhesions, BFT introduces a distinct biomechanical vulnerability. This represents a critical, complementary shift in MASH pathogenesis: integrating a state of sustained metabolic inflammation with accelerated structural impairment and advanced fibrotic progression.

### 2.2. Concurrent Barrier Disruption and Size-Dependent Systemic Translocation

A critical factor in ETBF-mediated pathology is its spatial expansion within the context of MASH-induced dysbiosis [21]. While accumulating metabolic factors (e.g., hydrophobic secondary bile acids) can inherently compromise junctional integrity [22], we hypothesize that BFT acts as a potent secondary effector in this process. Through the enzymatic cleavage of epithelial E-cadherin [6], BFT exacerbates the proteolytic disassembly of the physical mucosal barrier.

Beyond this enzymatic activity, BFT possesses a distinct size-dependent advantage for systemic dissemination. Unlike the bulky micellar aggregates typically formed by LPS (>100 kDa) [23], BFT is a compact, 20 kDa molecule [24]. This modest molecular mass facilitates efficient paracellular diffusion through the junctional gaps that have been progressively widened by both metabolic stress and its own proteolytic action.

The clinical significance of this translocation is further underscored by the remarkable persistence of ETBF colonization. In murine models, a single inoculation can result in colonization lasting the host’s lifespan [14], establishing a continuous, low-level secretion of the toxin. Crucially, recent evidence confirms that translocated BFT remains detectable in the systemic circulation for weeks [8]. We hypothesize that this sustained bioavailability establishes a state of chronic “proteolytic stress” on the hepatic parenchyma, allowing structural impairment to compound significantly over time.

### 2.3. Dual Mechanism of Hepatic Entry and Maintenance of Systemic Bioactivity

Upon reaching the hepatic sinusoids, we hypothesize that BFT enters the parenchyma through a dual-action mechanism. First, the compact size of the toxin (approx. 3–4 nm in diameter; 20 kDa) allows it to passively and efficiently diffuse through the large open fenestrae (50–200 nm) characteristic of Liver Sinusoidal Endothelial Cells (LSECs) [25].

Second, BFT may actively compromise the sinusoidal barrier integrity. Empirical evidence demonstrates that BFT functions as a direct protease against human endothelial barriers, inducing the specific cleavage of VE-cadherin and generating a distinct C-terminal fragment (CTF) [26]. From a biochemical perspective, this proteolytic event is fundamentally governed by enzyme–substrate specificity. Because the structural conformation of the VE-cadherin ectodomain is highly conserved [27,28], its susceptibility to BFT-mediated cleavage should theoretically be independent of the specific tissue location. Based on this conserved relationship, we postulate that BFT retains its proteolytic activity against VE-cadherin on LSECs. While LSECs are characterized by their unique fenestrae, the integrity of the sinusoidal barrier is further reinforced by junctional complexes, including tight junctions. Under healthy conditions, these structures are expected to limit the paracellular diffusion of BFT toward the junctional VE-cadherin. However, in the MASH-affected liver, the sinusoidal environment undergoes pathological remodeling, such as LSEC capillarization and inflammatory-mediated weakening of junctional stability [9,29]. We postulate that this pre-existing vascular fragility serves as a prerequisite, allowing the compact 20 kDa BFT to access and proteolytically target the VE-cadherin ectodomain, thereby exacerbating sinusoidal permeability.

**Figure 1:**
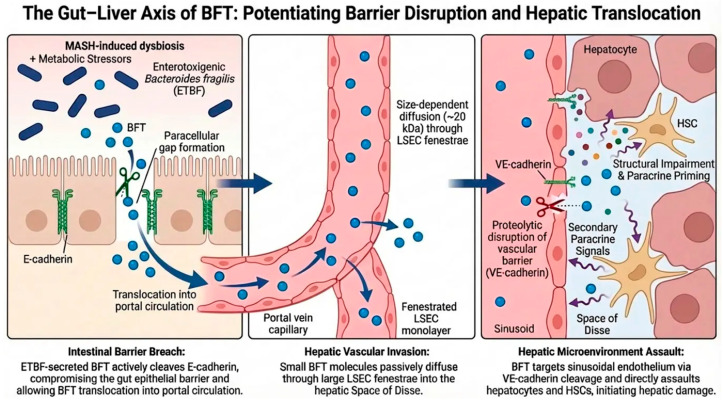
The Gut–Liver Axis of BFT: Concurrent Barrier Disruption and Hepatic Translocation. Known Mechanisms: Under MASH-induced dysbiosis, ETBF secretes BFT (~20 kDa), a metalloprotease with established E-cadherin cleavage activity. In the liver, the large fenestrae (50–200 nm) of the liver sinusoidal endothelial cells (LSECs) allow passive diffusion of compact molecules.

A critical concern regarding this active breach is whether BFT retains its biological activity during systemic transit. Crucially, in vivo models demonstrating that the intravenous tail-vein injection of BFT induces systemic sepsis strongly suggest that the toxin maintains an active proteolytic conformation despite exposure to the complex circulatory environment [26]. This functional persistence ensures that BFT reaching the Space of Disse remains fully capable of targeting parenchymal structures (Figure 1).

Proposed Hypothesis: Gut Barrier Breach: BFT acts concurrently with metabolic stressors to proteolytically amplify intestinal paracellular gap formation, facilitating massive systemic translocation. Hepatic Vascular Invasion: Upon reaching the liver, BFT may actively cleave VE-cadherin, further disassembling the sinusoidal barrier. Microenvironment Assault: Within the Space of Disse, BFT is hypothesized to acutely disrupt hepatocyte junctions, creating a pro-fibrogenic niche that potentiates Hepatic Stellate Cell (HSC) activation.

## 3. The Second Hit: Hepatocyte Junctional Collapse and Structural Impairment

The hepatic parenchyma, characterized by a highly organized assembly of polarized hepatocytes, serves as the liver’s primary metabolic engine [30]. Rather than acting solely as an inflammatory trigger, we hypothesize that systemic BFT functions as a targeted proteolytic effector. By driving the proteolytic disassembly of hepatocyte junctional complexes, BFT may precipitate a cascade of polarity loss and subsequent cellular stress.

### 3.1. Perisinusoidal Accumulation and Hepatocyte Targeting

Following its size-dependent translocation across the LSEC barrier (as detailed in Section 2), BFT gains direct access to the Space of Disse. In the context of MASH, altered hepatic hemodynamics and impaired sinusoidal clearance frequently promote the retention of macromolecules within this perisinusoidal space [29,31].

We hypothesize that this pre-existing microenvironmental dysfunction facilitates the prolonged accumulation of BFT at the basolateral membranes of hepatocytes. A significant hurdle for BFT within the hepatic parenchyma is the highly organized TJ barrier that maintains hepatocyte polarity and sequesters bile canaliculi [30]. Given that BFT lacks the direct enzymatic capacity to cleave tight junction proteins like ZO-1 or occludin [6,7], the toxin’s access to the basolaterally located E-cadherin is theoretically restricted in a healthy liver. We hypothesize that MASH-driven lipotoxicity and chronic inflammation act as ‘structural primers’ that partially de-anchor these apical TJ complexes. This localized architectural failure provides a ‘molecular window’ through which systemic BFT can infiltrate and execute its specific proteolytic strike on E-cadherin, triggering the subsequent collapse of canalicular networks and hepatic polarity.

Crucially, rather than operating independently, this localized proteolytic stress acts in tandem with the canonical inflammatory milieu. This pervasive accumulation sets the stage for the progressive destabilization of the hepatic architectural scaffold.

### 3.2. Biochemical Rationale: Acute Proteolytic Disruption of Hepatic E-Cadherin

BFT is a well-characterized metalloprotease known to cleave the extracellular domain of E-cadherin [6]. Given that intestinal and hepatic E-cadherin are identical gene products (encoded by *CDH1*), we postulate that hepatic E-cadherin shares a fundamental biochemical susceptibility to BFT-mediated cleavage. The physiological gravity of E-cadherin loss is underscored by in vivo models demonstrating that the genetic ablation of liver E-cadherin induces spontaneous steatohepatitis and carcinoma [32].

However, it is crucial to distinguish the action of BFT from genetic loss-of-function models. Rather than equating this process to a genetic “knockout,” we define BFT-mediated cleavage as an “acute proteolytic disruption.” Unlike genetic ablation—where cells may adapt or activate compensatory mechanisms over time—BFT induces a dynamic collapse of pre-established junctional architecture. We hypothesize that this acute enzymatic dismantling deprives hepatocytes of sufficient adaptation time, predisposing them to severe cellular stress (e.g., ER stress, anoikis). Consequently, the organized parenchyma transitions into a biomechanically vulnerable state, perfectly primed to cooperate with canonical MASH drivers.

### 3.3. Canalicular Structural Impairment and Localized Intrahepatic Cholestasis

We propose that a pivotal consequence of this proteolytic disassembly is the structural collapse of the bile canalicular network. In a healthy liver, E-cadherin-based adherens junctions are an essential prerequisite for maintaining the apical-basal polarity required for the initiation and maintenance of bile canaliculi [31,33].

Therefore, we postulate that the BFT-mediated destabilization of this junctional scaffold would severely compromise the canalicular architecture itself, rather than merely displacing specific transporters. This structural failure is likely to physically obstruct the directional flow of bile, precipitating a state of “localized intrahepatic cholestasis.”

**Figure 2:**
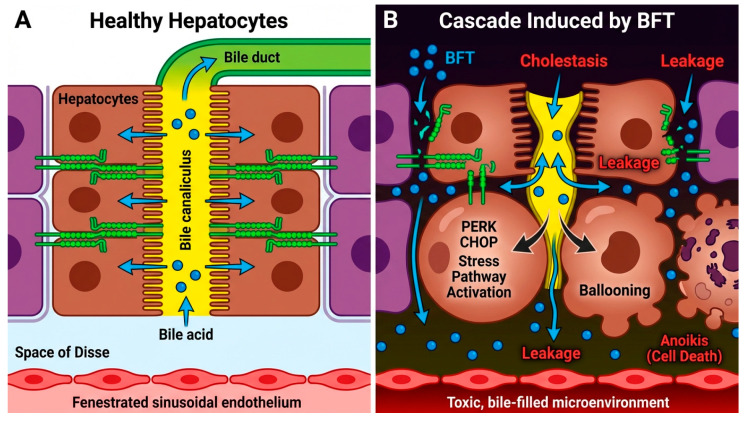
Hepatocyte Junctional Disassembly and BFT-Induced Cholestasis. (**A**) Known Physiology (Healthy Hepatocytes): Intact E-cadherin-based adherens junctions maintain strict apico-basal polarity, an essential prerequisite for bile canaliculi formation and directional bile secretion. (**B**) Proposed BFT-Mediated Pathogenic Cascade: The enzymatic cleavage of the E-cadherin ectodomain by systemic BFT is hypothesized to destabilize hepatocyte polarity, triggering a compounding cascade: (1) Cholestasis: Structural collapse of the bile canalicular network causes localized flow obstruction and the extravasation of lipotoxic bile acids. (2) Ballooning: Combined chemical stress and acute cytoskeletal failure drive the osmotic swelling of hepatocytes. (3) ER Stress and Anoikis: Loss of structural anchorage induces anoikis, while accumulating cleaved E-cadherin fragments may hyperactivate the PERK-CHOP stress pathway, accelerating cell death.

Consequently, hepatotoxic bile acids, having lost their compartmentalized conduit, would aberrantly diffuse into the paracellular space and the Space of Disse. Crucially, this leakage generates a severe lipotoxic secondary microenvironment. We hypothesize that these extravasated bile acids serve as a potent paracrine stimulus, acting together with existing inflammatory mediators to aggressively potentiate downstream Hepatic Stellate Cell (HSC) activation (Figure 2A,B).

### 3.4. Mechanistic Drivers of “Ballooning”: Coordinated ER Stress and Anoikis Vulnerability

Hepatocyte ballooning, a diagnostic hallmark of MASH, is proposed here as a morphological consequence significantly exacerbated by this proteolytic disassembly [33]. As anchorage-dependent cells, hepatocytes require continuous survival signals from intact cell–cell contacts. We hypothesize that the BFT-mediated cleavage of E-cadherin abruptly withdraws these signals, predisposing the cells to anoikis—a specialized form of apoptosis induced by the loss of structural attachment [34].

Concurrent with this structural destabilization, hepatocytes in a MASH microenvironment are inherently subjected to metabolic and lipotoxic stress. The sudden imperative to repair BFT-cleaved junctions by upregulating de novo E-cadherin synthesis imposes a severe, compounding burden on the endoplasmic reticulum (ER). Furthermore, the intracellular accumulation of cleaved cadherin fragments could overwhelm proteasomal clearance pathways, hyperactivating the Unfolded Protein Response (UPR) and the pro-apoptotic PERK-CHOP axis [35].

We postulate that this pathogenic convergence of anoikis signaling and UPR-driven organelle stress precipitates the acute cytoskeletal and osmotic failure that defines the classic “ballooned” hepatocyte morphology (Figure 2B).

### 3.5. Sinusoidal Remodeling and Pro-Fibrogenic Niche Formation

We postulate that the structural impact of BFT extends beyond parenchymal cells to profoundly alter the local microenvironment, creating a “pro-fibrogenic niche.” As detailed in Section 2, BFT’s targeted cleavage of VE-cadherin at the sinusoidal barrier is expected to accelerate vascular dysfunction. This endothelial remodeling, characterized by the pathological loss of fenestrae (capillarization), would severely impair metabolic exchange and favor the perisinusoidal retention of both BFT and damage-associated molecular patterns (DAMPs) [9,29].

The resulting pathological microenvironment—defined by localized hypoxia, extravasated lipotoxic bile acids, and accumulated cellular debris—would pervasively expose quiescent Hepatic Stellate Cells (HSCs) to a potent cocktail of primary stress signals [36,37].

Crucially, we hypothesize that this BFT-induced structural priming acts in powerful coordination with canonical inflammatory pathways. In the classical MASH paradigm, activated Kupffer cells and recruited macrophages drive fibrosis via the secretion of profibrogenic cytokines, most notably Transforming Growth Factor-beta (TGF-β) and Platelet-Derived Growth Factor (PDGF) [38,39]. By lowering the activation threshold of HSCs through biomechanical and lipotoxic stress, the BFT-induced niche profoundly amplifies their responsiveness to these traditional cytokines. Thus, BFT is envisioned not to replace, but to aggressively potentiate the established inflammatory drivers, creating a self-sustaining cycle of injury that accelerates advanced fibrotic progression.

## 4. Multimodal Orchestration of HSC Activation: Coordinated Junctional Disassembly

**Figure 3:**
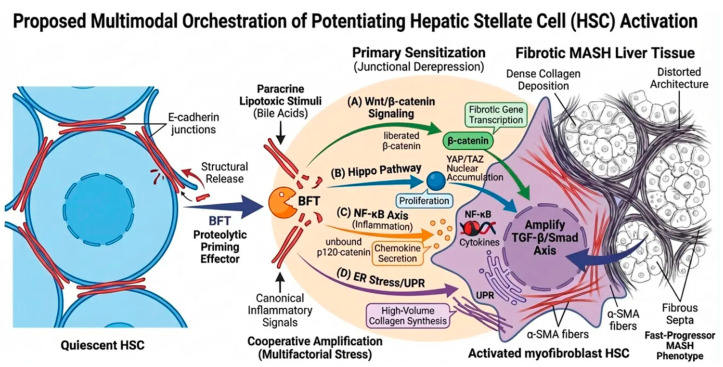
Proposed Multimodal Orchestration of Potentiated Hepatic Stellate Cell (HSC) Activation.

As established in Section 3, BFT creates a profoundly lipotoxic and pro-inflammatory microenvironment. However, we propose that BFT’s role extends beyond the generation of paracrine signals. Once within the Space of Disse, BFT also directly targets the adherens junctions on the surface of hepatic stellate cells (HSCs). Rather than acting as a solitary “catalytic switch,” BFT functions as a potent “Proteolytic Priming Effector.” By physically dismantling the E-cadherin complexes that actively repress fibrogenic mediators, BFT removes critical structural “brakes.” We hypothesize that this junctional shedding, when combined with the canonical pro-fibrogenic niche (e.g., TGF-β, DAMPs), cooperatively accelerates four cell-autonomous fibrogenic engines (Figure 3).

### 4.1. Mechanistic Drivers of Intracellular Reprogramming

In their quiescent state, HSCs paradoxically express E-cadherin to maintain adherens junctions, effectively sequestering pro-fibrogenic molecules at the plasma membrane [40]. We postulate that BFT-mediated E-cadherin shedding acts as a structural derepression, simultaneously priming multiple cell-autonomous pathways. First, the dissociation of the junctional complex may liberate β-catenin [38] and alleviate the contact-dependent inhibition of the Hippo pathway [41]. This dual release is hypothesized to drive the nuclear translocation of both β-catenin and YAP/TAZ, aggressively upregulating fibrotic and proliferative genes (e.g., Cyclin D1 and c-Myc) [42,43]. Second, the release of cytoplasmic p120-catenin could relieve the repression of NF-κB [42], transforming the HSC into an inflammatory hub that amplifies chemokine secretion (e.g., CCL2) [44]. Finally, the abrupt structural destabilization imposes a metabolic burden on the ER, activating the Unfolded Protein Response (UPR) to manage the massive secretory demands of de novo collagen synthesis [45,46].

Known HSC Biology: Quiescent HSCs express E-cadherin to maintain adherens junctions. The activation of HSCs into myofibroblasts typically involves multiple signaling cascades, including Wnt/β-catenin, Hippo (YAP/TAZ), NF-κB, and the Unfolded Protein Response (UPR), often driven by canonical signals like TGF-β and lipotoxicity.

Proposed BFT-Mediated Activation: We hypothesize that BFT functions as a “Proteolytic Priming Effector” by shedding HSC E-cadherin, acting as a structural release that primes cell-autonomous fibrogenic pathways.

Junctional Derepression: Liberated β-catenin (A) and YAP/TAZ (B) may translocate to the nucleus to drive fibrotic gene transcription and proliferation. Simultaneously, unbound p120-catenin could trigger NF-κB-mediated inflammation (C), while UPR is activated to support collagen synthesis (D).

Microenvironmental Amplification: This BFT-primed intracellular reprogramming is thought to be aggressively amplified by secondary paracrine stressors (e.g., extravasated lipotoxic bile acids) and canonical inflammatory signals, driving the “fast-progressor” MASH phenotype.

### 4.2. Microenvironmental Amplification via Paracrine Cholestatic Stress

Crucially, we propose that this BFT-primed intracellular reprogramming is profoundly amplified by the pathological microenvironment established in Section 3. The hepatotoxic bile acids aberrantly extravasating from the collapsed canalicular network act as a potent paracrine lipotoxic stimulus on adjacent HSCs. Extensive empirical evidence demonstrates that these cytotoxic bile acids (e.g., deoxycholic acid) can directly stimulate HSC proliferation and fibrogenesis by inducing oxidative stress and activating EGFR signaling pathways [47]. We hypothesize that this secondary cholestatic stress converges and cooperates with both the BFT-mediated junctional derepression and canonical inflammatory mediators. This multimodal convergence aggressively amplifies the canonical TGF-β/Smad signaling axis, effectively locking the hepatic microenvironment into a self-perpetuating cycle of advanced fibrotic progression (Figure 3).

## 5. Current Limitations and Future Perspectives

Despite the compelling biochemical rationale for the proposed role of *Bacteroides fragilis* toxin (BFT) in MASH progression, several critical gaps remain that necessitate cautious interpretation when extrapolating in vitro findings to complex in vivo systems.

### 5.1. Translating Biochemical Certainty to In Vivo Detection

Recent structural and in vitro studies have demonstrated that BFT directly and specifically cleaves the extracellular (EC) domain of E-cadherin [48], establishing unquestionable mechanistic proof of its proteolytic capability at the cellular level. However, a primary concern remains the scarcity of direct in vivo evidence demonstrating this active cleavage within the human hepatic parenchyma. We propose that this detection gap stems from profound technical hurdles rather than the absence of the mechanism:

- Transient Kinetics: BFT translocation may follow a “hit-and-run” mechanism, where the initial cleavage of the EC domain occurs rapidly during dysbiotic “blooms,” triggering downstream intracellular cascades (e.g., β-catenin release) that persist long after the toxin is degraded [49,50].
- Structural Homology: Furthermore, detecting intact BFT in situ is hindered by its structural homology within the metzincin superfamily. BFT shares highly conserved catalytic motifs with host metalloproteases (MMPs and ADAMs) [16]. This molecular mimicry complicates the generation of discriminatory antibodies due to cross-reactivity. Therefore, future research must shift focus from detecting the toxin itself to identifying stable “proteolytic footprints”—specifically, the soluble E-cadherin EC domain fragments shed into the serum—as diagnostic proxies for ETBF-associated hepatic injury [49,51].

### 5.2. Evolutionary Gaps in Liver-Specific Validation

While the direct cleavage of the E-cadherin EC domain is biochemically validated, liver-specific functional validation remains an ongoing challenge. The precise expression dynamics and functional sequestration of E-cadherin in quiescent versus activated hepatic stellate cells (HSCs) remain subjects of academic debate [40]. Our model posits that BFT-mediated “structural derepression” serves as a crucial priming event. However, quantifying the magnitude of this proteolytic priming relative to canonical drivers (e.g., TGF-β) within the highly specialized architecture of the liver—including the fenestrated Liver Sinusoidal Endothelial Cells (LSECs)—requires sophisticated gnotobiotic in vivo models. Furthermore, future research must explicitly investigate the sequential dynamics of this paracellular pathway, specifically evaluating how pre-existing tight junction impairment facilitates the deep tissue penetration of systemic BFT.

### 5.3. Integration into a Multifactorial Cooperative Framework

Crucially, despite its proven capability to directly dismantle junctional barriers, we do not propose BFT as a solitary driver of MASH. MASH is inherently multifactorial. Our hypothesis positions BFT as a “Proteolytic Priming Effector” that functions complementarily alongside established drivers such as LPS-TLR4 signaling and lipotoxicity [52]. The “fast-progressor” phenotype is likely determined by the combined convergence of BFT-induced structural destabilization (initiated by EC domain shedding) and pre-existing metabolic stressors.

## 6. Conclusions

This review proposes a model wherein enterotoxigenic *Bacteroides fragilis* (ETBF) contributes to the structural disruption of the gut–liver axis. Based on in vitro evidence of BFT-mediated E-cadherin cleavage, we hypothesize that this targeted proteolysis serves as a facilitator in MASH pathogenesis.

The proposed “dual-hit” on architectural integrity—involving the alteration of E-cadherin on hepatocytes/HSCs and VE-cadherin on LSECs—may compromise the biomechanical stability of the hepatic microenvironment. This structural modification is thought to act cooperatively with canonical inflammatory and metabolic pathways, potentially contributing to the accelerated disease progression seen in a subset of MASH patients.

To further evaluate this Cooperative Proteolytic Model, future research should incorporate longitudinal studies correlating fecal *bft* levels with specific serum proteolytic biomarkers. Understanding the ETBF–BFT axis as a complementary factor in MASH may eventually inform targeted therapeutic approaches, such as microbiome modulation or specific metalloprotease inhibition.

## Data Availability

No new data were created or analyzed in this study. Data sharing is not applicable to this article.

## Abbreviations

The following abbreviations are used in this manuscript:

ADAM	A Disintegrin and Metalloprotease
BFT	Bacteroides fragilis Toxin
CCL2	C-C Motif Chemokine Ligand 2
CDH1	Cadherin 1
CHOP	C/EBP Homologous Protein
CTF	C-Terminal Fragment
DAMPs	Damage-Associated Molecular Patterns
EC	Extracellular
EGFR	Epidermal Growth Factor Receptor
ER	Endoplasmic Reticulum
ETBF	Enterotoxigenic *Bacteroides fragilis*
HCC	Hepatocellular Carcinoma
HSC	Hepatic Stellate Cell
HSP47	Heat Shock Protein 47
IL-6	Interleukin 6
IRE1α	Inositol-Requiring Enzyme 1 alpha
kDa	Kilodalton
LPS	Lipopolysaccharide
LSEC	Liver Sinusoidal Endothelial Cell
MASH	Metabolic Dysfunction-Associated Steatohepatitis
MMP	Matrix Metalloproteinase
NF-κB	Nuclear Factor kappa-light-chain-enhancer of activated B cells
PDGF	Platelet-Derived Growth Factor
PERK	Protein Kinase R (PKR)-like Endoplasmic Reticulum Kinase
TAZ	Transcriptional Coactivator with PDZ-binding Motif
TGF-β	Transforming Growth Factor-beta
TLR4	Toll-Like Receptor 4
UPR	Unfolded Protein Response
VE-cadherin	Vascular Endothelial-cadherin
XBP1s	X-box Binding Protein 1 (spliced)
YAP	Yes-Associated Protein
